# The effectiveness and safety of Chinese medicines for the treatment of uveitis: A protocol for systematic review and meta-analysis

**DOI:** 10.1097/MD.0000000000020766

**Published:** 2020-06-26

**Authors:** Mengyu Han, Yang Chen, Luqi Nong, Ziqiang Liu, Yali Qin, Huan Meng, You Chen, Zhijun Wang, Ming Jin

**Affiliations:** a Beijing University of Chinese Medicine; b Department of Ophthalmology, China-Japan Friendship Hospital, Beijing; c State Key Laboratory of Ophthalmology, Zhongshan Ophthalmic Center, Sun Yat-sen University, Guangzhou, China.

**Keywords:** Chinese medicines, protocol, systematic review, uveitis

## Abstract

**Background:**

Uveitis is an inflammatory and heterogeneous ocular disorder and has a profound impact on patients’ life, work and family. There are substantial costs to the countries and individuals associated with treatment of the complications of uveitis and blindness. Conventional therapies did not lead to satisfactory outcomes for uveitis and are associated with substantial adverse events (AEs). Emerging evidences have proved the important value and potential prospect of Chinese medicines and its compound in uveitis. However, although Chinese medicines are widely used in uveitis, its therapeutic effect and safety are still controversial. It is, therefore, timely to perform an objective and normative systematic review to assess the efficacy and safety of Chinese medicines in treating uveitis on current research.

**Methods:**

The systematic review will include all of the randomized controlled trials (RCT) on the efficacy and safety of Chinese medicines for uveitis. A relevant literature search by sensitive search strategies was conducted using the following electronic databases from their inception to September 30, 2019: PubMed, Web of Science, EMBASE, the Cochrane Library, China National Knowledge Infrastructure (CNKI), Wanfang Database, China Science and Technology Journal database (VIP) and Chinese Biomedical Literature database (CBM). The strategy combines treatment terms and disease: that is, “Medicine, Chinese Traditional” (e.g., “Medicine, Chinese Traditional”, TCM, Traditional Chinese medicine, Zhong Yi Xue) and uveitis. We will also search registers of clinical trials, potential gray literature, and conference abstracts. There are no limits on language and publication status. The literature screening, data extraction, and quality assessment will be conducted by 2 reviewers independently. The reporting quality and risk of bias will be assessed by other two researchers. Best-corrected visual acuity (BCVA) and improvement in disease activity were assessed as the primary outcome. The secondary outcomes will include laboratory efficacy indexes, score changes in the National Eye Institute Visual Functioning Questionnaire 25 (NEI-VFQ 25), uveitis-related tissue damage or complications, concurrent requirement of corticosteroids, immunosuppressive drugs or biologics, and AEs of treatment. Meta-analysis will be performed using RevMan5.3 software provided by the Cochrane Collaboration.

**Results:**

This study will provide a comprehensive review based on current evidence of Chinese medicines treatment for uveitis in several aspects, including BCVA and improvement in disease activity, laboratory efficacy indexes, score changes in the NEI-VFQ 25, uveitis-related tissue damage or complications, etc.

**Conclusion:**

The conclusion of this study will provide evidence to determine whether Chinese medicines are an effective and safe intervention for patients with uveitis.

**Ethics and dissemination:**

It is not necessary to obtain ethical approval for this study, given that this protocol is for a systematic review. The systematic review will be published in a peer-reviewed journal, presented at conferences and will be shared on social media platforms.

**PROSPERO registration number:**

PROSPERO CRD42020153620.

## 1 Introduction

Uveitis is an inflammatory and heterogeneous ocular disorder, most commonly occurs in the working age population, which mainly contains the iris, the ciliary body and the choroid, or surrounding tissues (e.g., retina, sclera, and optic nerve)^[1]^ and is responsible for approximately 10% of blindness in western countries.^[2,3]^ The incidence and prevalence of uveitis differs based on age, anatomic location of the inflammatory process (anterior, intermediate, posterior uveitis, pan-uveitis), gender, histopathology (granulomatous, non-granulomatous), type of inflammatory process (acute, chronic, recurrent), and etiology (infectious, non-infectious).^[4]^ Current epidemiological data give yearly prevalence of uveitis of between 58 and 115 per 100000. The incidence is between 14 and 17 per 100,000.^[2,3,5]^ About 35% of patients with uveitis have significant visual impairment or legal blindness^[6,7]^ and its median age of presentation with uveitis is 36 years.^[8]^ In addition, studies indicate that it is increasing in incidence.^[2]^ A higher incidence of disease may be observed in Chinese and Japanese populations.^[9]^ There are also mounting concerns that juvenile idiopathic arthritis is the most common rheumatic disease in children and uveitis is possibly its most devastating extra-articular manifestation.^[10]^ The recognized criteria for the classification of uveitis is the Standardization of Uveitis Nomenclature (SUN) criteria, which included onset, duration and course of uveitis in the classification of the condition.^[11]^ Symptoms of uveitis depend on the parts of the eye affected, which were mainly recurrent ocular pain, photophobia, tears, blurred vision and red eyes. Most non-infectious causes appear to be autoimmune or autoinflammatory in nature, for example, uveitis is the most common extra-articular complication of Ankylosing spondylitis (AS), occurring in up to 50% of patients.^[12,13]^

Loss of visual function has a profound impact on patients’ life, work and family. There are substantial costs to the countries and individuals associated with treatment of the complications of uveitis and blindness. Besides, there is no cure available currently. Treatment is aiming at easing the symptoms, reducing and preventing inflammation, controlling the immune system, preserving and restoring vision and improving quality of life. The identification of an infectious cause of a particular uveitis will direct appropriate antimicrobial treatment, treatment is aimed at eradicating the pathogenic organism with appropriately targeted antimicrobial therapy.^[10,14]^ Conventional medicine commonly treats non-infectious uveitis by means of anti-inflammatory drugs, corticosteroids (systemic or local injection or implant), immunosuppressive drugs (such as mycophenolate mofetil, azathioprine and calcineurin inhibitors (such as tacrolimus and ciclosporin)) and biologics.^[1]^ However, these conventional therapies did not lead to satisfactory outcomes for some active uveitis and were associated with substantial adverse events (AEs).^[1,15]^

Chinese medicine, a significant component of Chinese preeminent traditional culture, is a collection of thousand years of medical practice experience. Chinese medicines has the advantages of multi-component, multi-pathway and multitarget synergies with fewer AEs and more therapeutic effects, compared with the exposure to single-target chemical drugs.^[16]^ Emerging evidences have proved the important value and potential prospect of Chinese medicines and its compound in uveitis. Jing et al^[17]^ constructed a Qinghuo Rougan Mingmu formula (QHRGF)-compound target-uveitis network, demonstrated that QHRGF attenuated local inflammation in experimental autoimmune uveoretinitis (EAU) rats by regulating natural killer T cells and inhibiting MAPK signal pathways. Tian et al^[18]^ proved Qingkailing injection can alleviate autoimmune uveitis in rats, inhibit the differentiation toward Th1 and Th17 effector cells and the relevant cytokines secretion. The therapeutic effect may also be regulated through increased secretion of interleukin (IL)-10. Longdan Xiegan Tang, another mixture of herbal extracts commonly used in traditional Chinese medicines, can efficiently alleviate the symptoms of EAU, inhibit the differentiation of uveitogenic CD4+T cells and reduce the expression of proinflammatory cytokines, including IFN-γ, IL-17 and TNF-α. Furthermore, Longdan Xiegan Tang promotes the production of IL-10 and accelerates the recovery of EAU.^[19]^ Traditional Chinese Herb Pairs, Angelica sinensis and Sophora flavescens, not only significantly inhibited the upregulation of NF-κB activation and the production of ICAM-1, IL-1β, TNF-α, iNOS, and COX-2 in rats of endotoxin-induced uveitis, but also suppressed maleic dialdehyde, polymorphonu-clear cells.^[20]^ Relevant clinical studies have also proved the actual clinical efficacy and advantages of Chinese medicines in the treatment of uveitis.^[21–23]^ However, although Chinese medicines is widely used in uveitis, its therapeutic effect and safety are still controversial. It is, therefore, timely to perform a systematic review to assess the efficacy and safety of Chinese medicines in treating uveitis on current research.

## 2 Methods

This protocol has been registered on PROSPERO (registration number: CRD42020153620).^[24]^ Our protocol will follow the Cochrane Handbook for systematic reviews of interventions and the preferred reporting items for systematic reviews and meta-analysis protocol (PRISMA-P) statement guidelines.^[25,26]^

### 2.1 Inclusion criteria for study selection

#### 2.1.1 Types of studies

We will consider only clinical randomized controlled trials (RCTs) of Chinese medicines in the treatment of uveitis. The current clinical trial results will be objectively integrated, which is conducive to the evaluation of the efficacy and safety of Chinese medicines for uveitis. We will exclude non-RCTs, quasi-RCTs, uncontrolled trials, reviews, case studies, case-controlled studies, qualitative studies, animal trials, and laboratory studies.

#### 2.1.2 Types of patients

Patients diagnosed as having uveitis will be included in the study. There will also be no restrictions based on other conditions, such as age, sex, race, educational or economic status, disease duration, and disease severity.

#### 2.1.3 Types of interventions

Patients in the experimental group were only treated with Chinese medicines, and the types and dosage forms of Chinese medicines prescriptions were not limited. Besides, western medicines and other treatment methods were not combined. Studies that with combination therapy fail to objectively evaluate the efficacy and safety of Chinese medicines will be excluded. The control interventions will include no therapy, placebo, western medicines, and other therapies.

#### 2.1.4 Types of outcome measures

##### 2.1.4.1 Primary outcomes

The primary outcomes of this review mainly include the following aspects:

(1) Best-corrected visual acuity (BCVA): measured according to a validated measure such as the ETDRS chart, Snellen chart or a similar tool, other measures of visual acuity would be considered if outcomes could be justified and validated in relation to accepted relevant standard measures.^[1]^
(2) Improvement in disease activity (e.g., vitreous haze score grade, anterior chamber cells grade)^[1,11,27]^

##### 2.1.4.2 Secondary outcomes

The secondary outcomes of this review mainly include the following aspects:

(1) Laboratory efficacy indexes: C-reactive protein (CRP), erythrocyte sedimentation rate, blood biochemical tests such as IgA, IgD, IgE, and IgM, and gamma-glutamyl transpeptidase.^[28]^
(2) Score changes in the National Eye Institute Visual Functioning Questionnaire 25 (NEI-VFQ 25)^[29]^
(3) Uveitis-related tissue damage or complications (e.g., cataract, macular edema, retinal vascular occlusion)^[1]^
(4) Concurrent requirement of corticosteroids, immunosuppressive drugs or biologics^[1]^
(5) AEs of treatment

##### 2.1.4.3 Security index

The safety was assessed by the occurrence of AEs. Any unexpected events that occurred during the studies will be recorded on an adverse event report form.

### 2.2 Search methods for the identification of studies

#### 2.2.1 Electronic searches

A relevant literature search by sensitive search strategies was conducted using the following electronic databases from their inception to September 30, 2019: PubMed, Web of Science, EMBASE, the Cochrane Library, China National Knowledge Infrastructure (CNKI), Wanfang Database, China Science and Technology Journal database (VIP) and Chinese Biomedical Literature database (CBM). The strategy combines disease and treatment terms: that is, “Medicine, Chinese Traditional” (e.g., “Medicine, Chinese Traditional”, TCM, Traditional Chinese medicine, Zhong Yi Xue) and uveitis. The search strategy for PubMed is listed in Table 1, which including all search terms, and other searches will be conducted based on these results. This will be appropriately adapted for search in the other databases. There are no limits on language and publication status.

**Table 1:**
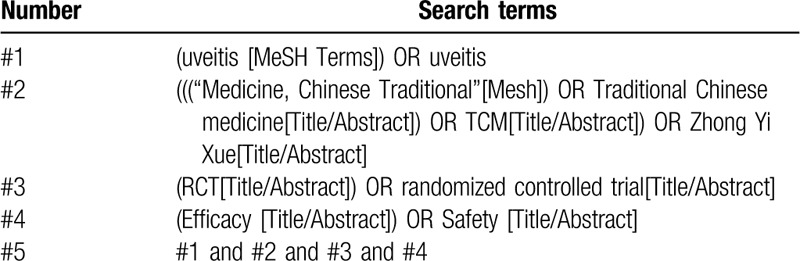
Search strategy used in PubMed database.

#### 2.2.2 Searching other resources

We will also search PROSPERO, the International Clinical Trials Registry Platform, ClinicalTrials.gov, dissertations, and gray literature to identify systematic reviews or clinical trials related to Chinese medicines and uveitis. Relevant journals and conference processes will be manual searched. We will also review papers and bibliographies included in the trials.

### 2.3 Data collection and analysis

#### 2.3.1 Selection of studies

**Figure 1:**
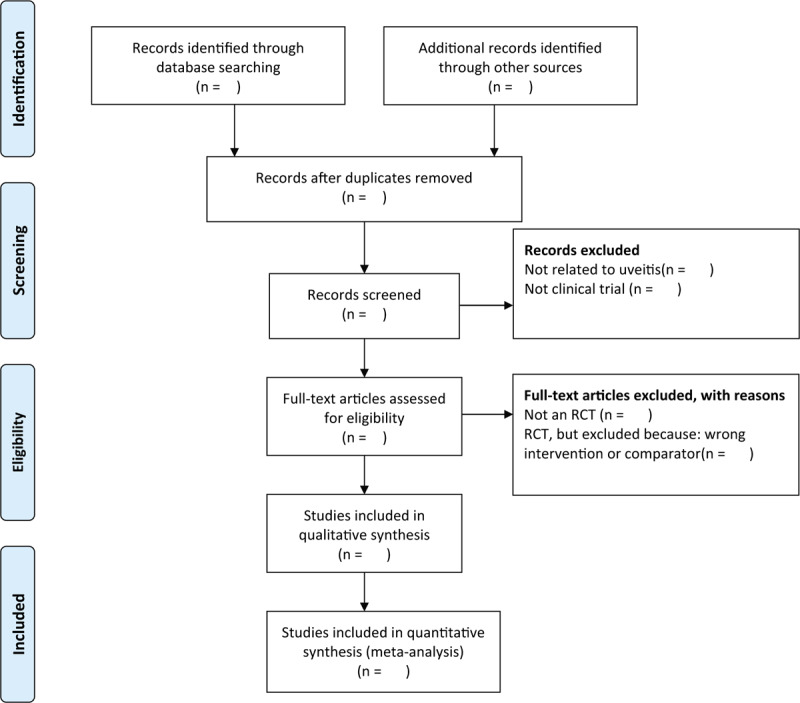
The PRISMA flow chart of the selection process. PRISMA = preferred reporting items for systematic reviews and meta-analysis protocol.

Two reviewers (MYH and YC) will independently browse the titles and abstracts of all of the retrieved records to distinguish and exclude any obviously irrelevant articles. We will select studies involved any form of Chinese medicines as the sole treatment or as a major therapy. Chinese medicines will be classed as the major therapy when the literature suggests that the frequency of application of Chinese medicines is higher and the time is longer than other intervention methods. Studies only related to human subjects will be included. Any disagreements will be resolved by discussion between the 2 authors and an arbiter (MJ). The study selection procedure is presented in a PRISMA flow chart (Fig. 1).

#### 2.3.2 Data extraction and management

Based on the inclusion criteria, a standard data collection form will be produced prior to data extraction. Search results will be entered into an EndNote X8 database and duplicate entries removed. Two authors (MYH and ZQL) will extract the data of interest from the eligible study and enter the following information in the data extraction sheet: The basic characteristics of each study (study type, author, title, source/journal, time of publication, country, hospital setting, study design); participants characteristics (average age, gender, sample size, inclusion and exclusion criteria, baseline situation); Interventions (type of Chinese medicines, randomization, allocation concealment, blinding methods, and duration and frequency); Comparators (no therapy, placebo, western medicines); Outcomes (measures, main outcomes, security indexes, and follow up); If funded, it will also be recorded. When the consensus on data extraction is not available through discussion, the third reviewer (MJ) will make a decision.

#### 2.3.3 Assessment of risk of bias

Two authors (YC and LQN) will independently evaluate the risk and bias using the Cochrane risk of bias assessment tool.^[30]^ The RevMan software program (V.5.3) will record the selected details of each study.^[31]^

#### 2.3.4 Measures of treatment effect

The risk ratio (RR) and 95% confidence interval (CI) will be used to analyze dichotomous data and measure the treatment effect. A weighted mean difference (WMD) or a standard mean difference with 95% CIs will be used to analyze continuous outcomes.

#### 2.3.5 Unit of analysis issue

We will only extract the 1st experimental period data of crossover trials to avoid carryover effects. Meanwhile, considering that there are multiple intervention groups in trials, we will combine all analogous groups into a single pairwise comparison to prevent a unit of analysis issue.

#### 2.3.6 Management of missing data

Reviewer (YLQ) will contact the appropriate author of the included trials for clarification via email and telephone if necessary. The missing data will be deleted, if there is no response from the author. In this case, this will be addressed in the discussion.

#### 2.3.7 Assessment of heterogeneity and data synthesis

We will use the complete case data as the analysis data. Heterogeneity will be tested with a standard Chi-square test.^[32]^ In order to quantify the impact of the statistical heterogeneity on the systematic review, the I^2^ value will be applied to calculate and present the heterogeneity degree. When *P* > .1, I^2^ < 50%, it is considered that there is no heterogeneity between the trials, and the fixed effect model will be used, otherwise, the random effect model will be adopted. All statistical analyses will be performed using RevMan5.3 software provided by the Cochrane Collaboration. Using the software to obtain forest plots and test the heterogeneity between the included studies. The grades of recommendation, assessment, development and evaluation will be used to assess the meta-analysis findings and determine the quality of evidence. Narrative comprehensive synthesis will be adopted, if meta-analysis is not possible due to lack of clinical studies or heterogeneity.

#### 2.3.8 Assessment of reporting biases

When 10 or more studies are included in a meta-analysis, we will assess funnel plot asymmetry for reporting biases and small study effects using Egger method.^[33]^ As funnel plot asymmetry does not necessarily suggest reporting bias, we will try to distinguish possible reasons for the asymmetry, including poor methodological quality and true heterogeneity of studies.

#### 2.3.9 Subgroup analysis

When heterogeneity is detected, a subgroup analysis will be conducted to judge the source of heterogeneity. The criteria for a subgroup analysis are as follows:

(1) Type of Chinese medicines therapies.
(2) Research quality.
(3) Participation population.
(4) Type of control interventions.
(5) Intervention frequency and duration.

#### 2.3.10 Sensitivity analysis

In the case of sufficient trials data, the risk of bias tool will be used to assess methodological quality. If low-quality articles are deleted, a second meta-analysis will be performed. The results and effect size of the two meta-analyses will be compared and discussed.^[34]^

## 3 Discussion

Uveitis is an inflammatory and heterogeneous ocular disorder, which can be divided into infectious and non-infectious.^[1,4]^ About 35% of patients with uveitis have significant visual impairment or legal blindness.^[6,7]^ Most non-infectious causes appear to be autoimmune or autoinflammatory in nature.^[12,13]^ Conventional therapies did not lead to satisfactory outcomes for uveitis and are associated with substantial AEs.^[1,15]^ Corticosteroids, as first-line treatment, have been the mainstay of uveitis for several years, which have a vital role in terms of “rescue” therapy. But their long-range use as a maintenance therapy is associated with many systemic and ocular complications, including gastric conditions, osteoporosis and fractures, mood disorders, skin conditions, cortico-induced diabetes, obesity, ocular conditions (including cataract) and cerebrovascular disease.^[1,14,15,35]^ Immunosuppressive drugs, the second-line treatment, have been widely used for uveitis in the following cases: to control inflammation in the condition of failure of or unresponsive to corticosteroids, and/or to take precautions against steroid-sparing agents.^[36,37]^ However, these agents lack the speed of onset and efficacy of corticosteroids and many are associated with the development of different, and equally limiting side effects, including gastrointestinal symptoms, cytopenia, and hepatotoxicity, with abnormal liver function observed in 15% of patients, hypertension, renal impairment, gingivitis, and hirsutism, bone marrow suppression, cutaneous malignancy, etc.^[15,38,39]^ At present, more biologics (TNF inhibitors, anti-IL-1β monoclonal antibody, anti-IL-6R monoclonal antibody, etc) are available for treatment of uveitis, aiming at regulating the inflammatory process, potentially by offering more specific targeted suppression of immune effector responses that damage tissue.^[40]^ The use of such drugs is also subject to many AEs, comprising increased risk of infections including TB and reactivation of chronic viral infections, malignancy, congestive and heart failure.^[41–45]^

Chinese medicines has the advantages of multi-component, multi-pathway and multitarget synergies with fewer AEs and more therapeutic effects, compared with the exposure to single-target chemical drugs.^[16]^ Emerging evidences have proved the important value and potential prospect of Chinese medicines and its compound in uveitis.^[17–23]^ However, although Chinese medicines is widely used in uveitis, its therapeutic effect and safety are still controversial. It is therefore timely to perform a systematic review to assess the efficacy and safety of Chinese medicines in treating uveitis on current research. The presented evidences were collected from RCTs with different evidence strengths to provide more comprehensive analysis. We expect that this systematic review will benefit patients with uveitis, clinicians, healthcare managers and policy-makers.

## Author contributions

MYH and Yang Chen conceived and designed the protocol, and MYH drafted the protocol manuscript. MYH developed the search strategy, with input from Yang Chen. MYH, HM and ZQL planned the data extraction. MYH, LQN and ZJW planned the quality appraisal of all included studies. MYH, ZQL, LQN, Yang Chen, HM, You Chen, YLQ, ZJW, and MJ critically revised the manuscript for methodological and intellectual content. All authors approved the final version.

**Conceptualization:** Mengyu Han, Ziqiang Liu, Yang Chen, Ming Jin.

**Data curation:** Mengyu Han, Ziqiang Liu, Huan Meng.

**Formal analysis:** Mengyu Han, Yang Chen.

**Project administration:** Mengyu Han, Ming Jin.

**Supervision:** Mengyu Han, Zhijun Wang, Ming Jin.

**Writing – review & editing:** Mengyu Han, Yang Chen.

## References

[1] Squires H Poku E Bermejo I A systematic review and economic evaluation of adalimumab and dexamethasone for treating non-infectious intermediate uveitis, posterior uveitis or panuveitis in adults. Health Technol Assess 2017;21:1–70. 10.3310/hta21680 PMC5723932 29183563

[2] Gritz DC Wong IG Incidence and prevalence of uveitis in Northern California-The Northern California Epidemiology of Uveitis Study. Ophthalmology 2004;111:491–500. 15019324 10.1016/j.ophtha.2003.06.014

[3] Suhler EB Lloyd MJ Choi D Incidence and prevalence of uveitis in veterans affairs medical centers of the pacific northwest. Am J Ophthalmol 2008;146:890–6. 19027424 10.1016/j.ajo.2008.09.014

[4] Tsirouki T Dastiridou A Symeonidis C A focus on the epidemiology of uveitis. Ocul Immunol Inflamm 2018;26:2–16. 27467180 10.1080/09273948.2016.1196713

[5] Acharya NR Tham VM Esterberg E Incidence and prevalence of uveitis: results from the Pacific Ocular Inflammation Study. JAMA Ophthalmol 2013;131:1405–12. 24008391 10.1001/jamaophthalmol.2013.4237

[6] Rothova A Suttorp-van Schulten MS Frits Treffers W Causes and frequency of blindness in patients with intraocular inflammatory disease. Br J Ophthalmol 1996;80:332–6. 8703885 10.1136/bjo.80.4.332 PMC505460

[7] Nussenblatt RB The natural history of uveitis. Int Ophthalmol 1990;14:303–8. 2249907 10.1007/BF00163549

[8] Çakar ⊠zdal MP Yazici A Tüfek M Epidemiology of uveitis in a referral hospital in Turkey. Turk J Med Sci 2014;44:337–42. 25536746 10.3906/sag-1302-132

[9] Hwang DK Chou YJ Pu CY Epidemiology of uveitis among the Chinese population in Taiwan: a population-based study. Ophthalmology 2012;119:2371–6. 22809756 10.1016/j.ophtha.2012.05.026

[10] Krishna U Ajanaku D Denniston AK Uveitis: a sight-threatening disease which can impact all systems. Postgrad Med J 2017;93:766–73. 28942431 10.1136/postgradmedj-2017-134891

[11] Jabs DA Nussenblatt RB Rosenbaum JT Standardization of uveitis nomenclature (SUN) working group. Standardization of uveitis nomenclature for reporting clinical data. Results of the first international workshop. Am J Ophthalmol 2005;140:509–16. 16196117 10.1016/j.ajo.2005.03.057 PMC8935739

[12] El Maghraoui A Extra-articular manifestations of ankylosing spondylitis: prevalence, characteristics and therapeutic implications. Eur J Intern Med 2011;22:554–60. 22075279 10.1016/j.ejim.2011.06.006

[13] Zeboulon N Dougados M Gossec L Prevalence and characteristics of uveitis in the spondyloarthropathies: a systematic literature review. Ann Rheum Dis 2008;67:955–9. 17962239 10.1136/ard.2007.075754

[14] Barry RJ Nguyen QD Lee RW Pharmacotherapy for uveitis: current management and emerging therapy. Clin Ophthalmol 2014;8:1891–911. 25284976 10.2147/OPTH.S47778 PMC4181632

[15] Jabs DA Rosenbaum JT Foster CS Guidelines for the use of immunosuppressive drugs in patients with ocular inflammatory disorders: recommendations of an expert panel. Am J Ophthalmol 2000;130:492–513. 11024423 10.1016/s0002-9394(00)00659-0

[16] Zhang YQ Mao X Guo QY Network pharmacology-based approaches capture essence of Chinese herbal medicines. Chinese Herb Med 2016;8:107–16.

[17] Jing C Sun Z Xie X Network pharmacology-based identification of the key mechanism of Qinghuo Rougan Formula acting on uveitis. Biomed Pharmacother 2019;9:109381. 10.1016/j.biopha.2019.109381 31542616

[18] Tian Q Bi H Cui Y Qingkailing injection alleviates experimental autoimmune uveitis in rats via inhibiting Th1 and Th17 effector cells. Biol Pharm Bull 2012;35:1991–6. 23123470 10.1248/bpb.b12-00449

[19] Tang K Guo D Zhang L Immunomodulatory effects of Longdan Xiegan Tang on CD4+/CD8+ T cells and associated inflammatory cytokines in rats with experimental autoimmune uveitis. Mol Med Rep 2016;14:2746. 27485320 10.3892/mmr.2016.5558

[20] Han C Guo J Antibacterial and anti-inflammatory activity of Traditional Chinese Herb Pairs, Angelica sinensis and Sophora flavescens. Inflammation 2012;35:913–9. 21976127 10.1007/s10753-011-9393-6

[21] Yang P Wang H Zhou H Therapeutic regimen in Vogt-Koyanagi-Harada syndrome. Zhonghua Yan Ke Za Zhi 2002;38:196–9. 12133384

[22] Zhou WY Zhang H Zhuang ZY Chinese medicine in the treatment of Behcet's disease's uveitis: a case report. Chin J Integr Med 2012;18:219–21. 22466948 10.1007/s11655-011-0949-y

[23] Davatchi F Chams-Davatchi C Shams H Behcet's disease: epidemiology, clinical manifestations, and diagnosis. Expert Rev Clin Immunol 2017;13:57–65. 27351485 10.1080/1744666X.2016.1205486

[24] Mengyu H You C LuQi N The effectiveness and safety of Chinese medicines for the treatment of uveitis: a protocol for systematic review and meta-analysis. PROSPERO 2020 CRD42020153620 Available from: https://www.crd.york.ac.uk/prospero/display_record.php?ID=CRD42020153620 10.1097/MD.0000000000020766 PMC7328941 32590753

[25] Shamseer L Moher D Clarke M Preferred reporting items for systematic review and meta-analysis protocols (PRISMA-P) 2015: elaboration and explanation. BMJ 2015;350:7647. 10.1136/bmj.g7647 25555855

[26] Moher D Shamseer L Clarke M Preferred reporting items for systematic review and meta-analysis protocols (PRISMA-P) 2015 statement. Syst Rev 2015;4:1. 25554246 10.1186/2046-4053-4-1 PMC4320440

[27] Denniston AK Holland GN Kidess A Heterogeneity of primary outcome measures used in clinical trials of treatments for intermediate, posterior, and panuveitis. Orphanet J Rare Dis 2015;10:97. 26286265 10.1186/s13023-015-0318-6 PMC4545540

[28] Chen Y Luo D Cai JF Effectiveness and safety of Glycyrrhizae Decoction for Purging Stomach-Fire in Behcet disease patients: Study protocol for a randomized controlled and double-blinding trail. Medicine (Baltimore) 2018;97:e0265. 29595687 10.1097/MD.0000000000010265 PMC5895409

[29] Schiffman RM Jacobsen G Whitcup SM Visual functioning and general health status in patients with uveitis. Arch Ophthalmol 2001;119:841–9. 11405835 10.1001/archopht.119.6.841

[30] Savović J Weeks L Sterne JA Evaluation of the Cochrane Collaboration's tool for assessing the risk of bias in randomized trials: focus groups, online survey, proposed recommendations and their implementation. Syst Rev 2014;3:37. 24731537 10.1186/2046-4053-3-37 PMC4022341

[31] Collaboration RTC. Review Manager Version 5.0. Copenhagen: The Nordic Cochrane Centre, The Cochrane Collaboration, 2008.

[32] Chen B Benedetti A Quantifying heterogeneity in individual participant data meta-analysis with binary outcomes. Syst Rev 2017;6:243. 29208048 10.1186/s13643-017-0630-4 PMC5718085

[33] Egger M Smith GD Schneider M Bias in meta-analysis detected by a simple, graphical test. BMJ 1997;315:629–34. 9310563 10.1136/bmj.315.7109.629 PMC2127453

[34] Shih KC Lun CN Jhanji V Systematic review of randomized controlled trials in the treatment of dry eye disease in Sjogren syndrome. J Inflamm (Lond) 2017;14:26. 29200970 10.1186/s12950-017-0174-3 PMC5698951

[35] Liu D Ahmet A Ward L A practical guide to the monitoring and management of the complications of systemic corticosteroid therapy. Allergy Asthma Clin Immunol 2013;9:30. 23947590 10.1186/1710-1492-9-30 PMC3765115

[36] Baker KB Spurrier NJ Watkins AS Retention time for corticosteroid-sparing systemic immunosuppressive agents in patients with inflammatory eye disease. Br J Ophthalmol 2006;90:1481–5. 16914474 10.1136/bjo.2006.097998 PMC1857545

[37] Kheir V Vaudaux J Guex-Crosier Y Review of the latest systemic treatments for chronic non-infectious uveitis. Expert Rev Ophthalmol 2016;11:111–33.

[38] Kaçmaz RO Kempen JH Newcomb C Cyclosporine for ocular inflammatory diseases. Ophthalmology 2011;117:576–84. 10.1016/j.ophtha.2009.08.010 PMC2830390 20031223

[39] Kremer JM Alarcón GS Lightfoot RW Jr Methotrexate for rheumatoid arthritis. Suggested guidelines for monitoring liver toxicity. American College of Rheumatology. Arthritis Rheum 1994;37:316–28. 8129787 10.1002/art.1780370304

[40] Takeuchi M A systematic review of biologics for the treatment of noninfectious uveitis. Immunotherapy 2013;5:91–102. 23256801 10.2217/imt.12.134

[41] Chung ES Packer M Lo KH Randomized, double-blind, placebo-controlled, pilot trial of infliximab, a chimeric monoclonal antibody to TNF-a, in patients with moderate-to-severe heart failure: results of the anti-TNF Therapy against Congestive Heart Failure (ATTACH) trial. Circulation 2003;107:3133–40. 12796126 10.1161/01.CIR.0000077913.60364.D2

[42] Lin J Ziring D Desai S TNFα blockade in human diseases: an overview of efficacy and safety. Clin Immunol 2008;126:13–30. 17916445 10.1016/j.clim.2007.08.012 PMC2291511

[43] Domm S Cinatl J Mrowietz U The impact of treatment with TNF-α antagonists on the course of chronic viral infections: a review of the literature. Br J Dermatol 2008;159:1217–28. 18945310 10.1111/j.1365-2133.2008.08851.x

[44] Salgado E Gomez-Reino JJ The risk of tuberculosis in patients treated with TNF antagonists. Expert SquiRev Clin Immunol 2011;7:329–40. 10.1586/eci.11.6 21595599

[45] Keystone EC Does anti-TNF-α therapy affect risk of serious infection and cancer in patients with rheumatoid arthritis? A review of longterm data. J Rheumatol 2011;38:1552–62. 21572154 10.3899/jrheum.100995

